# Supplementary material for: Small-Molecule Inhibitors of TIPE3 Protein Identified through Deep Learning Suppress Cancer Cell Growth In Vitro
Source: Cells. 2024 Apr 30;13(9):771. doi: 10.3390/cells13090771 (PMC11082981; doi:10.3390/cells13090771)
Supplement: Supplementary file 1 [file cells-13-00771-s001.zip › cells-2940469-supplementary.pdf]

Supplementary material section 1:

Detailed procedure of MD and metadynamics simulation

The initial protein-compound complexes were from the top score conformation Schrödinger docking. The ligand was edited by pymol software [51] to make it in the correct protonation state at pH 7.

To save the computational resources, we have carried MD for TIPE3-compound complexes by removing the residueID 110 to 129 region (FSSKSLALQAQKKILSKIAS, at the begin of N terminal) for simulation (this part helix is far away from the binding cavity). Metadynamics simulations can estimate binding free energy calculation to explore whether protein-ligand will bind in solution. Metadynamics relies on adding a bias potential to sample the free energy landscape along a specific collective variable of interest [49,50]. Note that the binding free energy calculations from metadynamics may only be suitable for detecting the general trend of binding in virtual screening.

The MD simulation was carried out by Gromacs with AMBER-99 force field [51,52]. The topology of the ligand and the partial charges of the ligand were generated by ACPYPE [53], which relies on Antechamber [54]. Firstly, we created a dodecahedron box and put the target-ligand complex at the center. A minimum distance from the protein to the box edge was set to 1 nm. We filled the dodecahedron box with TIP3P water molecules [55], and the counter ions were added to neutralize the total charge using the Gromacs program tool [56]. The long-range electrostatic interactions under the periodic boundary conditions were calculated with the Particle Mesh Ewald approach [57]. A cutoff of 10 Å was used for van der Waals non-bonded interactions. Covalent bonds involving hydrogen atoms were constrained by applying the LINCS algorithm [58].

We performed the energy minimization steps with a step-size of 0.001ns, 100 ps simulation with an isothermal-isovolumetric ensemble (NVT), and 10ns simulation with the isothermal-isobaric ensemble (NPT) for water equilibrium. After that, a 40ns NPT production run (step size 2 fs) was carried out. The Parrinello-Rahman barostat and the modified Berendsen thermostat were used for simulation with a fixed temperature of 308 K and a pressure of 1 atm. RMSD and hydrogen bond number of the trajectory were calculated using Gromacs tools.

The simulation continued using the metadynamics approach to explore the free energy landscape. We carried 40ns metadynamics simulation with Plumed [59] patched Gromacs. The protein-ligand complex's interface coordination number of atoms was used as a collective variable (CV). The protein-ligand interface coordination numbers correlate with the numbers of atom contact, and a larger coordination number usually indicates that the protein-ligand is binding. The coordination number C is defined as follows by Plumed:

$$C = \sum_{i \in A} \sum_{j \in B} S_{ij} \quad (1)$$

and

$$S_{ij} = \frac{1 - \left(\frac{r_{ij} - d_0}{r_0} \right)^n}{1 - \left(\frac{r_{ij} - d_0}{r_0} \right)^m} \quad (2)$$

In the simulation, n was 8, m was 12, d_0 was 0 nm, and r_0 was 0.25 nm. d_0 is a parameter of the switching function. r_{ij} is the distance between atom i and atom j. The degrees of contact between two groups of atoms can be estimated by the above function [60]. Metadynamics simulation for each protein-ligand system was performed for 40 ns. During the metadynamics simulation, Gaussian values were deposited every 1 ps with a height of 0.3 kJ/mol. The widths of the Gaussians were 5 for the coordination number. The free energy landscapes of the metadynamics simulations along the CV were generated by the Plumed program and plotted using Gnuplot [61].

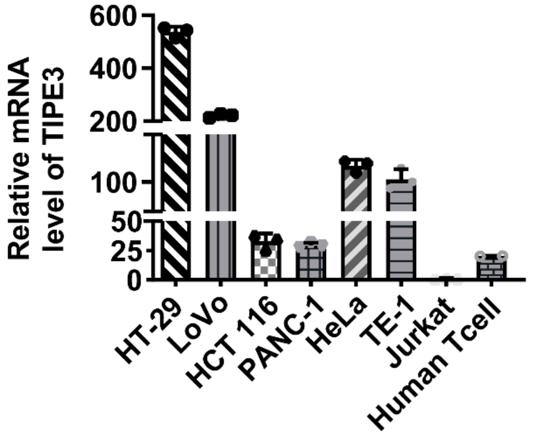

Supplementary Figure S1. Real-time PCR analyses of TIPE3 expression in different cell lines.

1. DeLano, W.L. PyMol: An open-source molecular graphics tool. *CCP4 Newsl. Protein Crystallogr.* **2002**, *40*, 82–92.
2. Hornak, V.; Simmerling, C. Generation of accurate protein loop conformations through low-barrier molecular dynamics. *Proteins* **2003**, *51*, 577–590. https:// doi.org/10.1002/prot.10363.
3. Hess, B.; Kutzner, C.; van der Spoel, D.; Lindahl, E. GROMACS 4: Algorithms for Highly Efficient, Load-Balanced, and Scalable Molecular Simulation. *J. Chem. Theory Comput.* **2008**, *4*, 435–447. https://doi.org/10.1021/ct700301q.
4. Sousa da Silva, A.W.; Vranken, W.F. ACPYPE-AnteChamber PYthon Parser interface. *BMC Res. Notes* **2012**, *5*, 367, https://doi.org/10.1186/1756-0500-5-367.
5. Wang, J.; Wang, W.; Kollman, P.A.; Case, D.A. Automatic atom type and bond type perception in molecular mechanical calculations. *J. Mol. Graph. Model.* **2006**, *25*, 247–260. https://doi.org/10.1016/j.jmgm.2005.12.005.
6. Jorgensen, W.L.; Chandrasekhar, J.; Madura, J.D.; Impey, R.W.; Klein, M.L. Comparison of simple potential functions for simulating liquid water. *J. Chem. Phys.* **1983**, *79*, 926–935. https://doi.org/10.1063/1.445869.
7. Van Der Spoel, D.; Lindahl, E.; Hess, B.; Groenhof, G.; Mark, A.E.; Berendsen, H.J. GROMACS: fast, flexible, and free. *J. Comput. Chem.* **2005**, *26*, 1701–1718. https://doi.org/10.1002/jcc.20291.
8. Darden, T.; York, D.; Pedersen, L. Particle mesh Ewald: An N·log(N) method for Ewald sums in large systems. *J. Chem. Phys.* **1993**, *98*, 10089–10092. https://doi.org/10.1063/1.464397.
9. Hess, B.; Bekker, H.; Berendsen, H.J.C.; Fraaije, J.G.E.M. LINCS: A Linear Constraint Solver for molecular simulations. *J. Comput. Chem.* **1997**, *18*, 1463–1472. https://doi.org/10.1002/(SICI)1096-987X(199709)18:12<1463::AID-JCC4>3.0.CO;2-H.
10. Tribello, G.A.; Bonomi, M.; Branduardi, D.; Camilloni, C., Bussi, G. PLUMED 2: New feathers for an old bird. *Comput. Phys. Commun.* **2014**, *185*, 604–613. https://doi.org/https://doi.org/10.1016/j.cpc.2013.09.018.
11. Williams, T.; Kelley, C.; Campbell, J.; Cunningham, R.; Denholm, D.; Elber, G.; Fearick, R.; Grammes, C.; Hart, L.; Hecking, L.; et al. Gnuplot 4.6. Softw. Man. 2012.
